# Persistent Sciatic Artery Aneurysm with Lower Limb Ischemia

**DOI:** 10.1155/2014/183969

**Published:** 2014-02-10

**Authors:** Gaurav Kesri, Jitendra Mangtani, Gaurav Kumar, Krishan Kumar Dangayach

**Affiliations:** ^1^Mahatma Gandhi Medical College and Hospital, RIICO Institutional Area, Sitapura, Jaipur 302022, India; ^2^Department of General Surgery, Mahatma Gandhi Medical College and Hospital, RIICO Institutional Area, Sitapura, Jaipur, Rajasthan 302022, India

## Abstract

Persistent sciatic artery is a very rare clinical entity. Those of us who have not seen the lesion regard this as a condition which is described in the literature through less than 200 cases. We report, here, a case of a 60-year-old female who presented to the surgical outdoor with complaints of a pulsatile gluteal swelling associated with ischemic changes in the ipsilateral lower limb. On Doppler and CT angiographic analysis, the patient was determined as having persistent sciatic artery aneurysm which was then managed by a combined surgical and endovascular approach. Ours is probably the first such case to be reported from India. The objective of this case report is to highlight the relevant embryology, the pathognomonic presenting features, the diagnostic dilemma, management, and complications associated with a case of persistent sciatic artery (PSA).

## 1. Introduction

PSA is a very uncommon embryological vascular aberration. The first description of such an anomaly was published as early as 1832 in the *Lancet* [1].The first description of aneurysm of the vessel, rupture of which resulted in the patients' death, was reported in 1864 [2]. The embryological basis and aberrant anatomy of this artery were elucidated in 1919 by reviewing a number of published case reports highlighting this condition [3, 4]. The largest clinical review of this condition was carried out by Ikezawa et al. in 1994 who reported an occurrence of 168 cases throughout the literature [5]. Throughout the literature the incidence of PSA has been estimated to be as low as 0.025–0.04% [6]. A tendency for aneurysm formation has been noted in 14.3–44% of such cases [7]. Besides this, the PSA is prone to atherosclerosis and distal embolization. The correlation of a painful gluteal swelling with persistent sciatic artery aneurysm requires a very high index of clinical suspicion and, as in our case, clinicians at a peripheral health care centre might end up ordering for fine needle aspiration cytology of the swelling, which could prove to be catastrophic.

## 2. Case Presentation 

**Figure 1:**
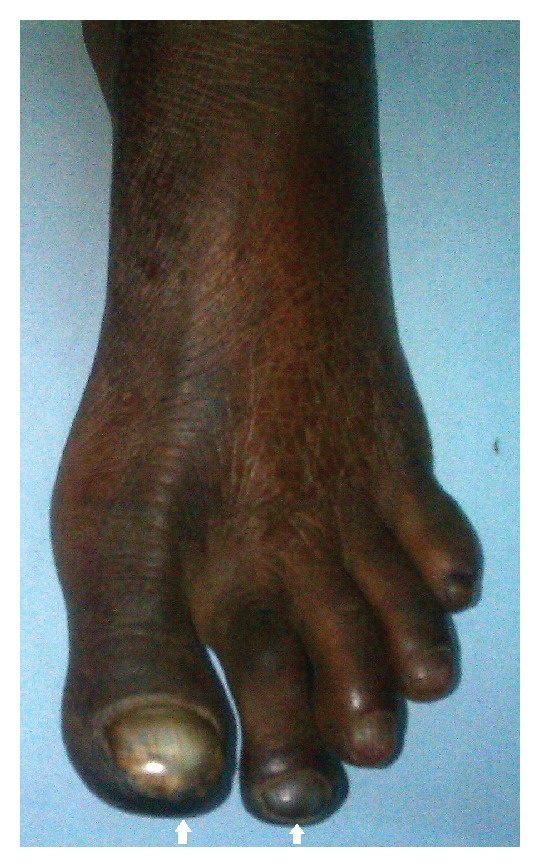
Left foot of the patient. The arrows depict gangrene of the 1st and 2nd toes. Also note the cyanosis of the rest of toes and ischemic changes on the skin of the foot.

A 60-year-old female presented with swelling in the left gluteal region for the last 2 years. The patient also complained of pain in the swelling for the past 6 months. Additionally the patient gave history of pain in the left lower limb for the past month with blackening of the toes of left lower limb for the past week (Figure 1).

The patient was found to be hypertensive on general examination. Fine needle aspiration cytology was attempted at a peripheral health centre, and on aspiration of blood, patient was urgently referred to our hospital.

Local examination revealed a swelling of about 6 × 6 cms in the left gluteal region. The swelling was cystic in consistency with ill-defined margins and was visibly pulsatile. Additionally, there were ischemic changes in the left lower limb with gangrene of the 1st and 2nd toes. Arterial examination revealed a cool left lower extremity with severely diminished femoral pulse, a normal popliteal pulse, and absent anterior tibial and dorsalis pedis pulse. The arterial pulses on the right side were normally palpable.

**Figure 2:**
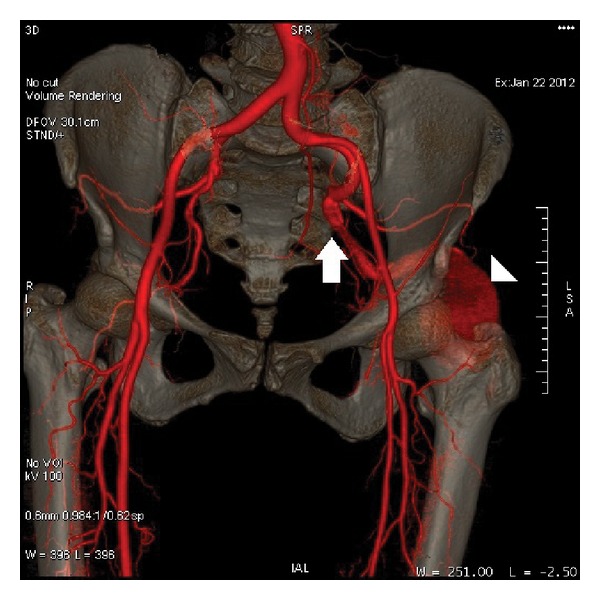
CT angiogram. The arrow depicts the abnormally large internal iliac artery. The arrowhead depicts the aneurysmal dilatation. Note the normal-sized internal iliac artery of the opposite side.

A duplex ultrasound of the left gluteal region revealed a deep-seated swelling with a marked arterial Doppler signature (see the video in the Supplementary Material available online at http://dx.doi.org/10.1155/2014/183969). A CT angiogram was ordered. The angiogram revealed a remarkably large left internal iliac artery with fusiform aneurysmal dilatation (Figure 2).

**Figure 3:**
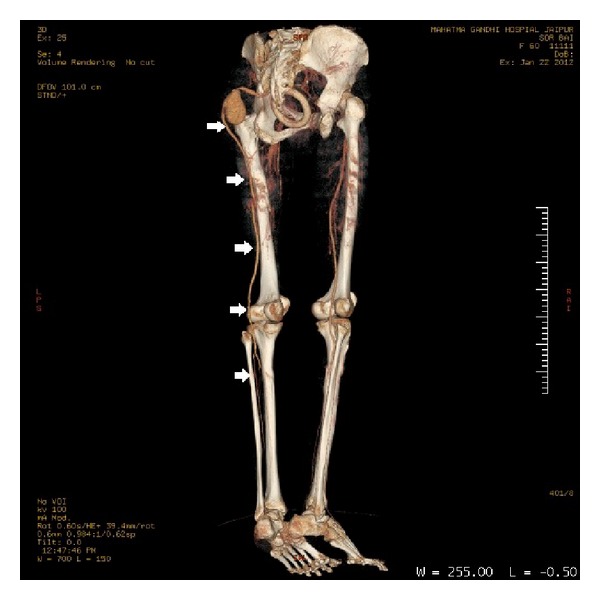
Volume rendering image of CT angiogram. The arrows show the course of persistent sciatic artery through the thigh and its distal run-off to the popliteal artery. Note that the popliteal artery of the opposite limb is supplied by the superficial femoral artery.

This artery was determined to be the PSA because of its continuity with the internal iliac artery, its course which was dilated and tortuous through the lateral thigh, and finally its distal run-off into the popliteal artery (Figure 3).

**Figure 4:**
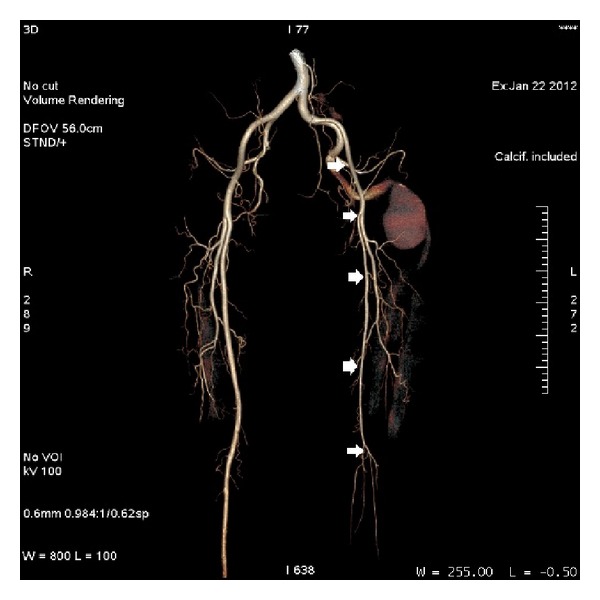
CT angiogram. The arrows depict the course of the external iliac artery and the superficial femoral system. Note that the femoral vessel is hypoplastic compared with the normal opposite limb and ends in the thigh.

The left superficial femoral system was hypoplastic and the superficial femoral artery ended distally in the thigh with no connection to the popliteal artery. The opposite limb had normal arterial anatomy (Figure 4).

The patient underwent a femoropopliteal bypass followed by a peripheral angiography and embolization of the left PSA aneurysm. After embolization, no flows were seen in the aneurysm. Left anterior tibial and posterior tibial arteries were normally palpable at the ankle. Due to the gangrene of the toes, patient underwent left forefoot amputation 1 week later. The postoperative period was uneventful and patient remains symptom-free with no evidence of ischemic changes till one year of followup.

## 3. Discussion

Though a very rarely encountered entity, the formation and persistence of a sciatic artery run a very interesting embryonic course. The primitive sciatic artery is given off from the dorsal root of the umbilical artery by as early as 6 mm embryo stage, to serve as the dominant supply for the developing lower limb bud [8–10]. Consequently, it runs dorsally to the growing lower limb all the way to the sole of the foot and forms the adult popliteal and peroneal vessels [4, 8, 9]. The umbilical artery gives off the external iliac artery, proximally to the origin of the sciatic artery [4, 10]. The adult arterial anatomy is defined by the occurrence of this significant development. The proximal umbilical artery will now form the adult common iliac artery, with the point of origin of external iliac artery serving as its bifurcation, hence resulting in sciatic artery now becoming the adult internal iliac artery. At the 12 mm embryo stage, the external iliac artery develops into the common and superficial femoral vessels, with the latter extending and terminating just above the knee [10]. The superior communicating ramus, which arises from the termination of superficial femoral artery, runs laterally to anastomose with the sciatic artery. This results in the double blood supply to the distal popliteal and peroneal vessels, both from the sciatic and superficial femoral vessels [10]. From the 18 mm embryo stage, the flow from the superficial femoral artery, through the superior communicating ramus, into the popliteal artery, dominates, resulting in the involution and discontinuity of the sciatic artery by the 22 mm embryo stage [10, 11]. The sciatic artery now persists only as the adult inferior gluteal artery [4, 8, 10].

**Figure 5:**
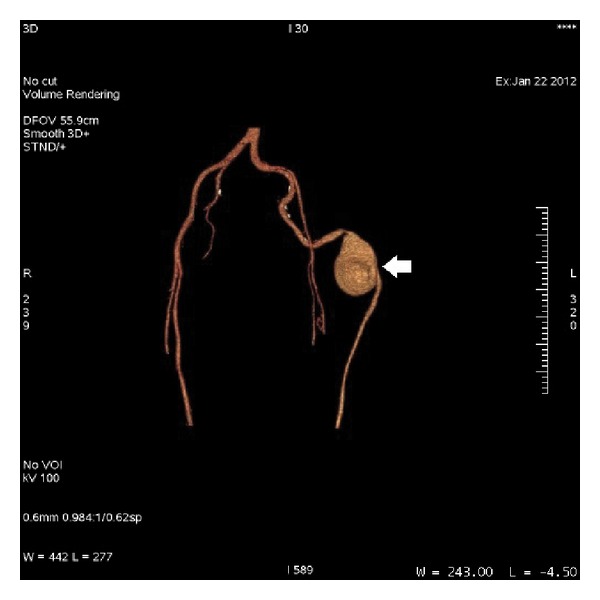
CT angiogram. The arrow shows the complete type of persistent sciatic artery with aneurysmal dilatation.

The failure of the superficial femoral artery to establish itself as the dominant supply of the lower limb results in the persistence of the sciatic artery and its classification as the complete and incomplete types [7, 9, 10]. The incomplete type is defined as the existence of a hypoplastic persistent sciatic artery along with the dominant superficial femoral vessel. The complete type entails the existence of a dominant persistent sciatic artery along with a hypoplastic superficial femoral system which only provides collateral supply to the lower limb [5, 9, 12]. Based on the above definitions, our patient can be concluded as having a complete type of persistent sciatic artery (Figure 5).

PSA has been reported in age range of 6 months to 85 years with a slight male preponderance [10, 13]. Bilaterality has been reported in almost 50% of the cases [11]. Most of these patients are diagnosed when lower limb symptoms result and when a high degree of clinical suspicion regarding this vascular anomaly is kept. The presenting symptoms like pain in the lower limb, numbness, and motor impairment result from compression of the nerves which lie adjacent to this aberrant artery in the thigh [2, 9, 10]. Atherosclerotic changes and aneurysm formation are common complications of PSA and are seen in up to 44% of the cases [5, 7, 8, 12].

A pulsating gluteal swelling with lower limb symptoms and/or ischemic changes in the ipsilateral limb is pathognomonic of persistent sciatic artery aneurysm. The pathophysiology of aneurysm is yet unknown although authors have postulated theories like congenital absence of elastic elements in the arterial wall and exposure of this artery to repeated episodes of trauma in hip as its cause [11]. The thrombosis of the aneurysm and resultant distal microemboli results in the ischemic changes and limb loss, unless promptly treated. Doppler ultrasonography is the investigation of choice and can confirm the presence of an aneurysm [11]. CT angiography remains the gold standard investigation as it can delineate the lower limb arterial system, define the aneurysm formation, and describe the degree of intraluminal thrombosis [8, 11, 12, 14]. Under no circumstances should fine needle aspiration cytology or any similar procedure be undertaken as the results could be disastrous.

This condition can be adequately dealt by surgical and endovascular intervention. Though Fung et al. have reported a case of persistent sciatic artery aneurysm managed conservatively with warfarin, a definitive surgical intervention should be undertaken before any significant limb loss occurs [12, 14]. It must be noted that most of such patients have complete type of PSA aneurysm and hence the vascular supply of the lower limb is almost completely dependent on this aberrant artery [5, 11]. Hence, a vascular reconstruction procedure is necessitated. Femoropopliteal bypass is the preferred method as the risk of graft compression is less [5, 7–9, 11, 12, 14]. The aneurysm can then be obliterated using gelfoam or coil embolisation [12, 14]. An incomplete type of PSA aneurysm can be adequately managed by stenting or embolisation of the aneurysm as the dominant vascular supply is achieved by the superficial femoral system.

## 4. Conclusion

PSA is a rare aberration and diagnosis can only be achieved by a CT angiogram read by a clinician who is well versed with this anomaly. Early management can result in preventing significant limb loss. As in our case diagnosis was made by CT angiography and the treatment followed a combined surgical and endovascular approach and though the patient underwent forefoot amputation, the rest of the limb was adequately revascularized by the femoropopliteal bypass and the patient remains symptom-free till one year of followup.

## Supplementary Material

The video shows a duplex scan of the swelling showing a deep seated swelling with a arterial signature.
